# Disentangling the effects of age and mild traumatic brain injury on brain network connectivity: A resting state fMRI study

**DOI:** 10.1016/j.nicl.2020.102534

**Published:** 2020-12-22

**Authors:** M. Bittencourt-Villalpando, H.J. van der Horn, N.M. Maurits, J. van der Naalt

**Affiliations:** University of Groningen, University Medical Center Groningen, Department of Neurology AB51, 9700RB Groningen, The Netherlands

**Keywords:** Brain connectivity, fMRI, mTBI, Aging, Resting-state

## Abstract

**Introduction:**

Cognitive complaints are common shortly after mild traumatic brain injury (mTBI) but may persist up to years. Age-related cognitive decline can worsen these symptoms. However, effects of age on mTBI sequelae have scarcely been investigated.

**Methods:**

Fifty-four mTBI patients (median age: 35 years, range 19–64 years, 67% male) and twenty age- and sex-matched healthy controls were studied using resting state functional magnetic resonance imaging in the sub-acute phase. Independent component analysis was used to identify intrinsic connectivity networks (ICNs). A multivariate approach was adopted to evaluate the effects of age and group on the ICNs in terms of (static) functional network connectivity (FNC), intensities of spatial maps (SMs) and time-course spectral power (TC).

**Results:**

We observed significant age-related changes for a) FNC: changes between 10 pairs of ICNs, mostly involving the default mode (DM) and/or the cognitive-control (CC) domains; b) SMs: intensity decrease in clusters across three domains and intensity increase in clusters across two domains, including the CC but not the DM and c) TC: spectral power decrease within the 0–0.15 Hz range and increase within the 0.20–0.25 Hz range for increasing age within networks located in frontal areas, including the anterior DM. Groups only differed for TC within the 0.065–0.10 Hz range in the cerebellar ICN and no age × group interaction effect was found.

**Conclusions:**

We showed robust effects of age on connectivity between and within ICNs that are associated with cognitive functioning. Differences between mTBI patients and controls were only found for activity in the cerebellar network, increasingly recognized to participate in cognition. Our results suggest that to allow for capturing the true effects related to mTBI and its effects on cognitive functioning, age should be included as a covariate in mTBI studies, in addition to age-matching groups.

## Highlights

• Aging affects brain network connectivity more strongly than mTBI.
• Effects of aging are most evident in the default mode and cognitive domains.
• mTBI might alter the frequency spectrum of the cerebellar network.

## 1 Introduction

Traumatic brain injury (TBI) is one of the most important causes of morbidity and mortality in adults (Carroll et al., 2014). Mild TBI (mTBI) accounts for 85% of the cases (Levin and Diaz-Arrastia, 2015). Cognitive and/or emotional complaints are common within the first weeks after mild traumatic brain injury (mTBI) and may persist for months to years in a subgroup (≈20%) (Levin and Diaz-Arrastia, 2015). Age-related cognitive decline can worsen these symptoms (Moretti et al., 2012). Although older age has been identified as an independent predictor of worse outcome after mTBI (Jacobs et al., 2010), few studies have investigated the effects of age on mTBI sequelae and the mechanism underlying this apparently interactive phenomenon remains unknown (McDonald et al., 2012, Thompson et al., 2006).

One way to capture brain mechanisms underlying changes in behavior is to study brain networks that subserve functions such as vision, cognition or movement control. Since it has been identified that brain networks exhibit consistent patterns across healthy participants, there has been growing interest in functional magnetic resonance imaging (fMRI) analyses that explore brain networks in the search for biomarkers of diseases. Several studies have reported that brain connectivity changes with age (see Ferreira and Busatto, 2013, Sala-Llonch et al., 2015 for reviews) and evidence has accumulated that there are alterations in the connectivity of brain networks after mTBI (see Mayer et al., 2015, Puig et al., 2020, Sharp et al., 2014 for reviews). In this study, we hypothesize that age affects the brain functioning and that its effect on brain connectivity can be disentangled from the effect of mTBI itself.

Brain networks can be analyzed at different levels. In this functional MRI (fMRI) study we investigate large-scale intrinsic connectivity networks (ICNs) as their interactions are relevant for high-level cognitive function (Seeley et al., 2007). ICNs are composed of clusters of voxels (Smith et al., 2013) that show temporally correlated blood oxygen level dependent (BOLD) responses and they can be grouped into functional domains according to their functional and anatomical roles (Allen et al., 2014, Du et al., 2020). Independent component analysis (ICA) is a data-driven approach that can be used to identify components that correspond to ICNs based on fMRI data. Based on identified ICNs, subsequent between-network functional connectivity analyses can provide information on how brain areas interact with each other based on pairwise correlation analysis of the temporal BOLD signal (time course) of ICNs. Within-network connectivity analyses can provide information about the network’s integrity by analyzing changes in the intensity of spatial maps and on the time course spectra of ICNs.

A number of ICNs and their respective domains are widely recognized, where the default mode network (DMN) is one of the most studied ICNs. The DMN is highly active when an individual is at rest as opposed to being engaged in a task. The DMN has two main cores: the anterior DMN, based in the ventral medial prefrontal cortex (vmPFC) and the posterior DMN, based in the posterior cingulate cortex (PCC), precuneus and the lateral parietal cortex (Raichle, 2015, Uddin et al., 2009). According to the literature, the vmPFC acts as a mediator in the interplay between emotional processing and cognitive functioning (Broyd et al., 2009, Gusnard et al., 2001) and both vmPFC and PCC are associated with introspective, self-referential processes (Gusnard et al., 2001). Fox et al. (2005) demonstrated that the activation in a set of ICNs that typically increases their activation when a person is engaged in a task (“task-positive network”) is anticorrelated to activation in ICNs of the DMN and that this anticorrelation pattern is preserved at rest. Since this apparent intrinsic organization of the brain has been identified, where the DMN plays a central role, investigating alterations in ICN patterns that occur with healthy aging or disease and how they relate to changes in behavior or cognitive performance emerged as a research topic of high interest (Broyd et al., 2009). For that reason, several studies on the mTBI population investigated altered connectivity patterns both between and within the DMN and other ICNs using ICA (Zhou et al., 2012) or other techniques such as seed-based analysis (Iraji et al., 2015) in search for insight into the underlying mechanisms of mTBI-related sequelae. Although the DMN (domain) is of particular interest, we will analyze its central role in whole brain connectivity in an exploratory fashion instead of pre-selecting areas of interest. The ICNs of the DMN have been consistently identified in resting state studies and, therefore, we expect that they will be among the ICNs revealed by fMRI data analysis. Thus, we will firstly identify ICNs using a data-driven approach and then we will group them according to the functional domains they belong to. The results will be discussed with a particular focus on the DMN.

Here, we use group independent component analysis (GICA; Calhoun et al., 2001) to identify the ICNs in a group of mTBI patients and healthy controls (HCs) and we use group information guided independent component analysis (GIG-ICA; Du and Fan, 2013) for the back-reconstruction of subject specific ICNs. GICA is one of the data-driven techniques that can be used to identify the components of brain networks (that are associated with functional aspects of the brain) and it has been widely used to make group inferences based on fMRI data. GIG-ICA achieves high reliability in back-reconstruction of subject specific ICNs (Du et al., 2017). Following this parcellation-approach, we assess changes in two aspects of FC: a) (static) between-network functional network connectivity (FNC) (Jafri et al., 2008) and b) within-network connectivity evaluated in terms of two complimentary measures: intensities of spatial maps activation (SMs) and power spectra of ICN time courses (TCs).

In summary, to get a better understanding of the neurological processes involved in the early recovery stages after mTBI and their possible interactions with aging, we aim to investigate age- and mTBI-related alterations in functional connectivity between and within the ICNs identified in our sample population. Together, changes in these two variables will allow to better understand the mechanisms behind cognitive complaints after mTBI.

## 2 Methods

### 2.1 Study participants

We studied fifty-four patients (median age: 35 years (IQR: 23–52), range 19–64 years, 36 male) with mTBI and 20 healthy participants (median age: 30 years old (IQR: 26–49), range 18–61 years, 14 male), whose data were obtained as part of a larger prospective multicentre follow-up study (UPFRONT study; van der Horn et al., 2016a, van der Horn et al., 2016b, van der Horn et al., 2017, van der Naalt et al., 2017). This sample cohort has been analyzed in previous studies (van der Horn et al., 2016a, van der Horn et al., 2016b, van der Horn et al., 2017). Patients were included at the University Medical Center Groningen, the Netherlands (a level 1 trauma center) between March 2013 and February 2015. The diagnosis of mTBI was based on a Glasgow Coma Scale score of 13–15 and/or loss of consciousness ≤30 min and/or post-traumatic amnesia up to 24 h (Vos et al., 2012). Healthy controls (HCs) were recruited via social contacts and advertisements. The HCs did not have any history of TBI or other neurological or psychiatric diseases and did not suffer from current psychiatric or neurological conditions. MTBI patients and HCs were group matched for age and sex (Van Der Horn et al., 2017).

The UPFRONT study was approved by the local Medical Ethics Committee of the UMCG; written informed consent was obtained from all participants. All procedures were performed according to the declaration of Helsinki.

### 2.2 Patient subgroups

A 21-item post-traumatic questionnaire (de Koning et al., 2016), derived from the Rivermead Post-concussion Symptoms Questionnaire (RPQ; King et al., 1995), was administered to the patients two weeks after the injury. The interval between the questionnaire’s administration (two weeks after injury) and the fMRI acquisition (four weeks after injury) is mainly related to the time required for planning for the fMRI scan. As previously described (van der Horn et al., 2016a, van der Horn et al., 2016b), the presence of post traumatic complaints (PTCs) was defined by self-reporting at least three complaints (regardless of severity). Consequently, two subgroups of patients were defined for presence (PTC-present) or absence (PTC-absent) of PTCs.

### 2.3 fMRI acquisition

A 3.0T Philips Intera MRI scanner (Philips Medical Systems, Best, The Netherlands) equipped with a 32-channel SENSE head coil was used for image acquisition. A high-resolution transversal T1-weighted sequence image was made for anatomical reference (repetition time [TR] 9 ms, echo-time [TE] 3.5 ms, flip angle 8°, field of view [FOV] 256 × 232 × 170 mm, reconstructed voxel size 1 × 1 × 1 mm). For resting-state imaging, T2*-weighted echo planar imaging volumes were acquired with slices aligned in the anterior commissure (AC)-posterior commissure (PC) plane and recorded in descending order (TR 2000  ms, TE 20 ms, FOV 224 × 224 × 136.5 mm, reconstructed voxel size 3.5 × 3.5 × 3.5 mm).

The patients’ fMRI resting state data were acquired at approximately four weeks (median: 33 days, range: 22–69 days) post-injury. All participants (both patients and controls) were instructed to close their eyes and to stay awake (duration: 10 min, 300 volumes).

### 2.4 fMRI preprocessing

Statistical Parametric Mapping (SPM12 Wellcome Department University College London, London, England) implemented in Matlab (version R2017b; MathWorks, Natick, MA) was used. The preprocessing followed the same pipeline previously used (Van Der Horn et al., 2017), which consisted of slice timing correction, image realignment to the first functional image, co-registration of functional images with individual participants’ T1-weighted images, normalization using a diffeomorphic nonlinear registration tool (DARTEL) (isotropic voxels of 3x3x3mm) to the Montreal Neurological Institute [MNI] template and smoothing (8 mm full-width at half maximum [FWHM] Gaussian kernel). The first five volumes out of a total of 300 volumes from each participant were excluded from the analysis to ensure T1 equilibrium.

### 2.5 Group independent component analysis

The Group ICA fMRI Toolbox (GIFT^1^; version 4.0b) was used to perform group-level spatial component analysis (Calhoun et al., 2001). The preprocessed fMRI data from 74 participants were decomposed into independent components (ICs) using group-information guided independent component analysis (GIG-ICA) (Du and Fan, 2013). The number of ICs (*N_c_*) was estimated using the minimum description length (MDL) criteria (Li et al., 2007). Subject-specific data reduction using two-step principal component analysis (PCA) first reduced the data to 100 principal components followed by group data reduction retaining *N_c_* ICs. The Infomax ICA algorithm (Bell and Sejnowski, 1995) was repeated 20 times using the built-in ICASSO^2^ tool, which is used to estimate the stability and quality of the ICs. Additional parameters for ICASSO were bootstrapping with randomized initial condition, minimum cluster size of 16 components (0.8*20 ICA repetitions) and maximum cluster size of 20 components (same value as the number of ICA repetitions) (Himberg et al., 2004). The quality of components was quantified as the quality index (I_q_; range 0–1) and the minimum I_q_ for inclusion of the component in the analysis was set at 0.9. The *N_c_* ICs from the best performing run were used as templates for subject-specific back-reconstruction using GIG-ICA (Du et al., 2014, Du and Fan, 2013). We identified the ICs considered to be ICNs, as opposed to physiological artefacts, by visually inspecting their aggregate spatial maps and their average power spectra. The identification of the ICNs was done by authors MBV and HJvdH independently. In accordance with previously published literature, the classification of components as ICNs was based on the following criteria: a) exhibiting peak activations primarily in gray matter, b) low spatial overlap with known vascular, ventricular motion and susceptibility artefacts and c) time courses dominated by low frequency fluctuations (Cordes et al., 2000). Differences were discussed until consensus was reached.

### 2.6 Connectivity assessment

For the set of *C* selected ICNs, we assessed three different but complimentary aspects of functional connectivity: (static) functional network connectivity (FNC; Jafri et al., 2008), related to the connectivity between networks; intensity of spatial maps activations (SMs), related to the degree of coactivation within a network; and power spectra of the ICN time course (TC), related to the level of coherent activity within a network (Allen et al., 2011). All measures of connectivity were calculated using the MANCOVAN toolbox^3^ (Allen et al., 2011) that is implemented in GIFT. We included sex and motion correction for head movement in the scanner as nuisance covariates. To reduce excessive statistical testing and the chance of spurious findings, we followed a multivariate approach (Allen et al., 2011).

FNC was estimated as the Pearson’s correlation of pairs of TCs (Allen et al., 2011, Jafri et al., 2008). Subject-specific TCs were detrended and despiked using 3dDespike^4^, then filtered using a fifth-order Butterworth low-pass filter with a high-frequency cut-off of 0.15 Hz. The variance associated with the motion parameter covariates was regressed out. For FNC statistical analysis, correlations were transformed to z-scores using Fisher’s transformation: *z* = atanh(FNC), as implemented in the MANCOVAN toolbox (Allen et al., 2011).

The ICN SMs were thresholded based on voxelwise t-statistics to limit the statistical analysis to voxels with strong and consisted activation across participants, as explained in (Allen et al., 2011). The threshold for voxel selection was set at t>μ+4σ.GIG-ICA automatically generates Z-scored ICs (Du and Fan, 2013).

Spectra were estimated for the detrended subject-specific TCs using the multi-taper approach as implemented in Chronux^5^, with the time-bandwidth product set to 3 and the number of tapers set to 5 (Mitra and Bokil, 2009). For all spectral analyses, spectra were log-transformed to normalize the highly skewed power distribution. For each of the *N* subjects, we thus have *C* power spectra with b = 257 elements (frequency bins) each for the frequency range 0–0.25 Hz.

### 2.7 Statistical analysis

For testing differences between (sub)groups in log(age) and sex, we used independent T-tests and Chi-Square tests, respectively. Age was first log-transformed to normalize its slightly skewed distribution. For testing differences between (sub)groups in sex, education level and interval between injury and fMRI scan, we used Chi-Square tests.

The design matrix included three covariates of interest: group as a categorical variable (mTBI patient or HC), age as a continuous variable and the interaction term group by age. In addition, we included two nuisance covariates: sex as a categorical variable (male or female) and average framewise displacement (FD; in mm) (Power et al., 2012) as a continuous variable representing head motion in the scanner. Sex was included because previous studies with HCs found small effects for sex on the outcome measures (Allen et al., 2011). FD was first log-transformed to normalize its slightly skewed distribution.

A second model was built to investigate differences between the subgroups PTC-present and PTC-absent and to include the interval between the accident and the scan (in days) as well as the GCS score as nuisance covariates. The second model was built because these two covariates are not applicable for HCs. The design matrix of the second model and its respective results can be found in the Supplementary Material (Sections A and D).

We used a multivariate model selection strategy based on multivariate analysis of covariance (MANCOVA) as implemented in the MANCOVAN toolbox. The purpose of this procedure is to assess to what extent each covariate explains the variance in each of the functional connectivity measures for selection of important covariates before performing the univariate tests. The MANCOVAN implementation tests the explained variance of each predictor for the multivariate response and performs backward selection of the model terms using a stepwise selection method using the mSTEPWISE algorithm. For more details on the MANCOVAN implementation, please refer to (Allen et al., 2011) and to Supplementary Material (Section B).

Univariate t-tests on SMs, TC power spectra and FNC were corrected for multiple comparisons at an α = 0.05 significance level using false discovery rate (FDR; Genovese et al., 2002) correction.

For FNC results, in case of main effects for age, we calculated the Pearson correlation coefficients (*r_AGE_*) to measure the strength of the linear relationship between age and FNC using the “corr” function implemented in Matlab. The results were visualized using scatter plots.

For SM results, in case of main effects for age, z-scores were averaged over the total number of voxels (*N*_*v*_) with significant effects of the same sign (positive or negative). We calculated the Pearson correlation coefficients (*r_AGE_*) to measure the strength of the linear relationship between age and (average) z-score using the “corr” function implemented in Matlab. The results were visualized using scatter plots.

For TC power spectra results, in case of main effects for age, log(power) was averaged over the total number of frequency bins (*N_b_*) with effects of the same sign (positive or negative). We calculated the Pearson correlation coefficients (*r_AGE_*) to measure the strength of the linear relationship between age and (average) TC power spectra using the “corr” function implemented in Matlab. The results were visualized using scatter plots. Additionally, we calculated the average TC power spectra (of the affected ICNs) for the younger and the older participants based on median split. In case of main effects for group, we calculated the average TC power spectra (of the affected ICNs) per group (mTBI and HCs) and subgroup (PTC-present and PTC-absent).

## 3 Results

The summary of participant characteristics per group of participants is presented in Table 1. Independent samples T-tests did not indicate any significant differences for log(age). Chi-square tests did not indicate significant differences for sex nor for education level between groups.

**Table 1:** – Participant characteristics per group.

	HC (N = 20)		mTBI patients (N = 54)	
	Median	Range	Median	Range
**Age (years)**	30 (27–48)	18–61	35 (23–51)	19–64
**Education level**^a^	6 (5–7)	5–7	6 (5–6)	2–7
**Interval injury to scan** (days)	–	–	33 (28–42)	22–69
**Sex**	**N**	**%**	**N**	**%**
Male	14	70	36	67
Female	6	30	18	33
**GCS**	**–**	**–**	**N**	**%**
15	–	–	29	54
14	–	–	18	33
13	–	–	7	13

a Education level was based on a Dutch classification system, according to Verhage (1964), ranging from 1 to 7 (highest). Abbreviations: GCS = Glasgow Coma Score.

The summary of mTBI patients’ characteristics per subgroup is presented in Table 2. The PTC-present subgroup contained more females than the PTC-absent subgroup (χ^2^ = 7.78, p < 0.01). Independent samples T-tests did not indicate any significant differences for log(age). Chi-square tests did not indicate significant differences for education level nor for interval between injury and fMRI scan between groups.

**Table 2:** mTBI patient characteristics per subgroup.

	mTBI PTC-present (N = 34)		mTBI PTC-absent (N = 20)	
	Median	Range	Median	Range
**Age** (years)	35 (23–50)	19–63	34 (23–57)	20–64
**Education level**^a^	6 (5–7)	(4–7)	6 (5–6)	(2–7)
**Interval injury to scan** (days)	33 (29–42)	22–62	33 (25–41)	22–69
**Sex**	**N**	**%**	**N**	**%**
Male	18	53	18	90
Female	16	47	2	10
**GCS**	**N**	**%**	**N**	**%**
15	15	44	14	70
14	15	44	3	15
13	4	12	3	15

a Education level was based on a Dutch classification system, according to Verhage (1964), ranging from 1 to 7 (highest). Abbreviations: GCS = Glasgow Coma Score.

### 3.1 Group independent component analysis and ICNs

**Fig. 1:**
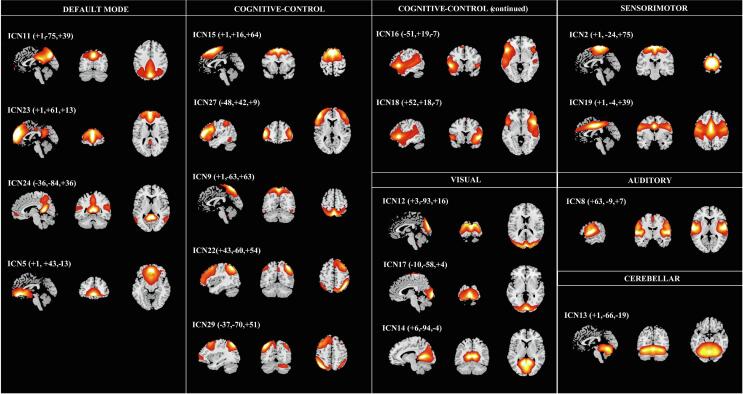
Spatial maps of the 18 intrinsic connectivity networks identified as belonging to functional domains, thresholded at z-score > 1.

The estimation using MDL criteria resulted in *N_c_* = 30 ICs. The final quality index (*Iq*) of all ICs was above 0.95, according to ICASSO tool estimation. Of the *N_c_* ICs extracted, we identified *C* = 18 ICNs that could be grouped into seven functional domains. The SMs of the 18 ICNs are shown in Fig. 1. The final set included four ICNs for the Default-Mode Network domain (DM), seven for the cognitive-control domain (CC), three for the visual domain (VIS), two for the sensorimotor domain (SMO), one for the auditory domain (AUD), and one for the cerebellar domain (CB).

### 3.2 Multivariate results

**Fig. 2:**
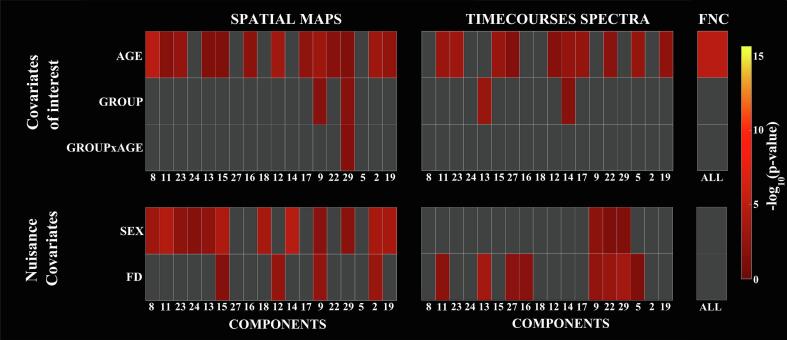
Results from the multivariate tests showing the significance of the covariates of interest and nuisance predictors for intensity of spatial maps, TC power spectra and FNC. Gray squares represent model terms that were not retained in the backward selection process (α = 0.05).

Fig. 2 illustrates the results from the multivariate tests representing the significance of the model terms age, group, group × age, sex and FD in predicting the outcome variables SMs, TC power spectra and FNC for the 18 identified ICNs. Age was retained as a significant predictor for all of the three connectivity measures. Sex was retained as significant predictor for SMs of several ICNs and for TC spectra of a few ICNs, supporting its incorporation as a nuisance covariate for measures of connectivity within networks. Group was found to be a weak significant predictor for the SMs and TC spectra of a few ICNs, but not for the FNC correlation matrix. The interaction group × age was found to be a weak significant predictor only for the SM of one ICN. FD was retained for several ICNs when predicting TC spectra and for some ICNs when predicting SMs, suggesting residual motion artefacts on ICN TCs.

### 3.3 Univariate results

Lastly, we aimed to identify which ICN pairs in the FNC correlation matrix are associated with age; which ICN regions (voxels) are associated with age, group and/or group × age and for which ICN spectral bins of the TC spectra power is associated with age and group. Therefore, univariate tests were performed on the covariates of interest for the terms that were retained in the backward selection step.

#### 3.3.1 FNC

**Fig. 3:**
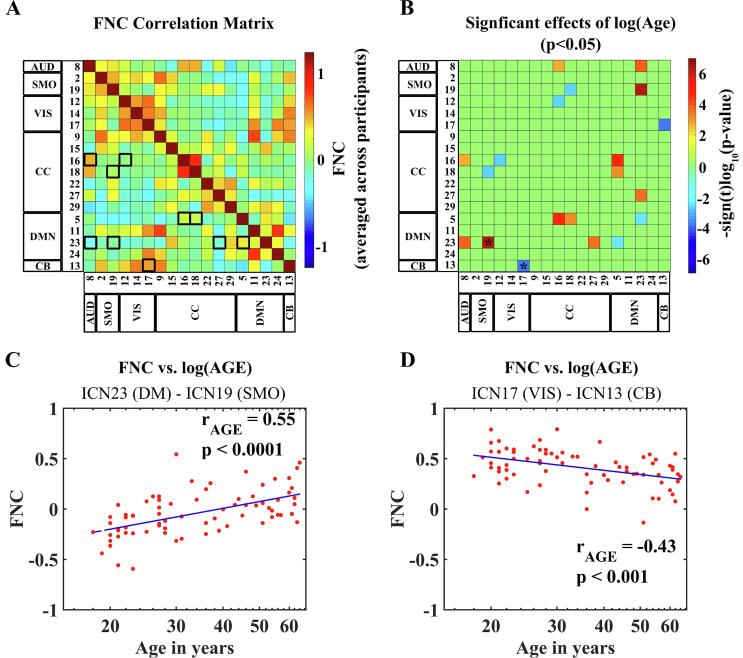
Effects of age on (static) functional network connectivity (FNC) between intrinsic connectivity networks (ICNs). (A): FNC matrix showing the pairwise correlations between ICN TCs (FNC) averaged across all participants. Black squares and rectangles highlight ICN pairs for which significant effects for log(Age) were found. (B): The matrix displays the significance and direction of the effects of age for each pairwise correlation (p < 0.05, FDR-corrected). (C, D): Scatterplots depicting how FNC between pairs of ICN TCs changes for increasing age based on two examples from the significant results displayed in (B): (C) is an example of positive age-related correlation (***r_AGE_***) between pairwise FNC and age, based on the pair ICN23 (DM) and ICN19 (SMO). (D) Is an example of negative age-related correlation (***r_AGE_***) between pairwise FNC and age, based on the pair ICN17 (VIS) and ICN13 (CB). The examples selected for the scatterplots (C) and (D) are highlighted in the FNC matrix (B) with asterisks. Age is presented in the scatterplots (C) and (D) on a log-scale.

Fig. 3 illustrates the effects of age on FNC between ICNs.

The matrix in Fig. 3A presents the FNC correlation between pairs of ICNs, averaged across all participants. To facilitate the visualization of results, we highlighted the 10 ICN pairs for which significant effects for log(Age) on FNC were found with black squares or rectangles. These significant results are depicted in Fig. 3B, where the FNC matrix displays the significance and sign (positive or negative) of the effects of age for each pairwise correlation. From a total of 10 ICN pairs, we chose one example of the most extreme positive and negative linear relationship between FNC and log(age) to depict in scatterplots (Figs. 3C, D).

The first scatterplot (Fig. 3C) shows an example of a positive linear relationship between log(age) and FNC for the pair ICN23 (anterior DM) and ICN19 (SMO). The correlation coefficient (*r*_*AGE*_ = 0.55, p < 0.0001) indicates moderate explained variance.

The second scatterplot (Fig. 3D) shows an example of a negative linear relationship between log(age) and FNC for the pair ICN17 (VIS) and ICN13 (CB). The correlation coefficient (*r*_*AGE*_ = −0.43, p < 0.001) also indicates moderate explained variance.

The scatterplots with the results from the remaining eight ICN pairs are given in in the Supplementary Material (Section C1, Fig. S1).

In total, we found 10 ICN pairs for which FNC was significantly correlated to log(age). Nine out of the 10 pairs involved one ICN from the DM (more specifically ICN23 or ICN5) or from the CC (more specifically ICN16, ICN18 or ICN27).

#### 3.3.2 Spatial map intensities

**Fig. 4:**
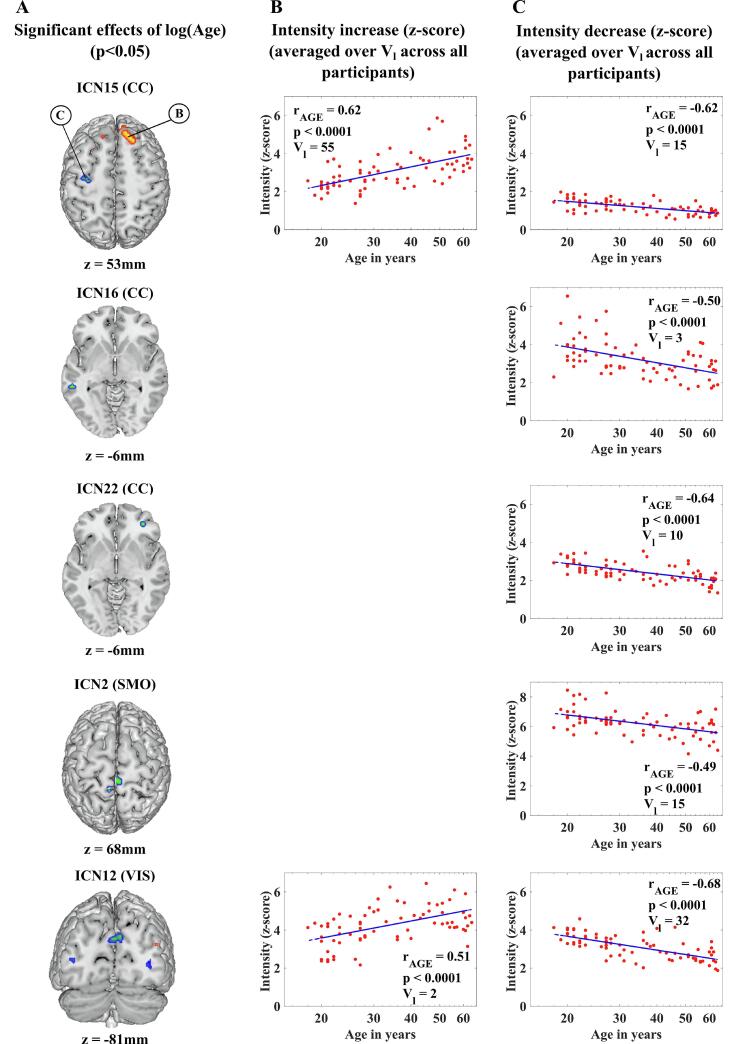
Effects of age on SM intensities. (A): Significant effects of age for each ICN SM (p < 0.05, FDR-corrected) in a representative slice. The top panel indicates an example of a cluster with significant SM intensity increase for increasing age (B in the circle) and an example of cluster with significant SM intensity decrease for increasing age (C in the circle). (B;C): Scatterplots depicting how SM intensities change for increasing age. Age is presented in the scatterplots on a log-scale.

The effects of age on SM intensities are shown in Fig. 4. The z-scores for which the univariate test statistics exceeded the FDR threshold (Fig. 4A) were averaged over voxels of the same sign (positive or negative). The number of voxels (*V_l_*) corresponds to the total number of voxels that contributed to the averaging of the displayed data.

Increases in SM intensities with age were found only in a two ICNs and the largest clusters were found in ICN15 (CC; *V_l_* = 55; *r*_*AGE*_ = 0.62, p < 0.0001).

Decreases in SM intensities with age were found in five ICNs across three domains, specifically ICNs 15, 16 and 22 (CC), ICN12 (VIS) and ICN2 (SMO). The largest clusters were found in ICN12 (VIS; *V_l_* = 32; *r*_*AGE*_ = 0.68, p < 0.0001).

#### 3.3.3 Timecourse spectra

**Fig. 5:**
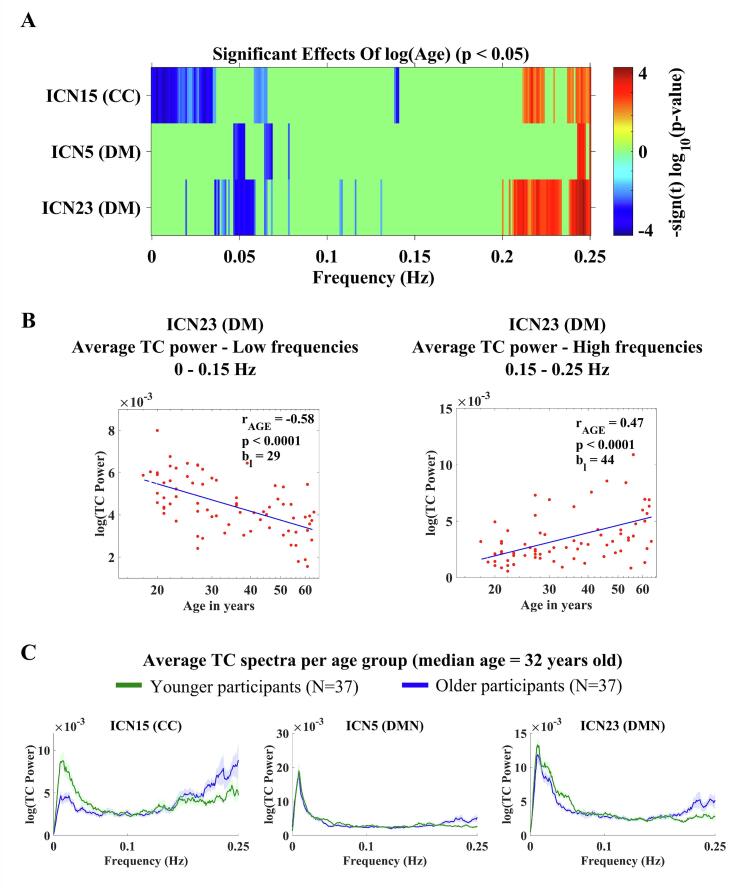
Effects of age on TC power spectra. (A): Effects of age for each IC TC power spectrum; the color bars display their significance and direction (p < 0.05, FDR-corrected). (B): Scatterplots depicting the significant results of changes in TC for increasing age for ICN23 (DM). (C): Line plots of the average TC power spectra of ICNs 5, 15 and 23 for younger and older participants based on median split (median age = 32 years old). The line plots show mean log(power) ± 1SE.

For low frequencies, below 0.15 Hz, TC power decreased significantly with age, for ICNs 5, 15 and 23 (p < 0.05, FDR-corrected). For these same ICNs, TC power increased significantly with age for higher frequencies, above 0.15 Hz (p < 0.05, FDR-corrected). These results are illustrated in Fig. 5A.

The power values where the univariate test statistics exceeded the FDR threshold (Fig. 5A) were averaged over frequency bins of the same sign (positive or negative). The number of frequency bins (*N_b_*) corresponds to the total number of bins that contributed to the averaging of the displayed data. The scatterplots (Fig. 5B) show the results for ICN23 (anterior DM), where we found a negative linear correlation between log(age) and average log(power) for low frequencies (*r*_*AGE*_ = −0.58, p < 0.0001) and a positive linear correlation between log(age) and average log(power) for high frequencies (*r*_*AGE*_ = 0.47, p < 0.0001). The scatterplots with the results from ICN5 and ICN15 can be found in the Supplementary Material (Section C2, Fig. S2).

The average TC power spectra (for the ICNs 5, 15 and 23) for younger and older participants are presented in Fig. 5C.

**Fig. 6:**
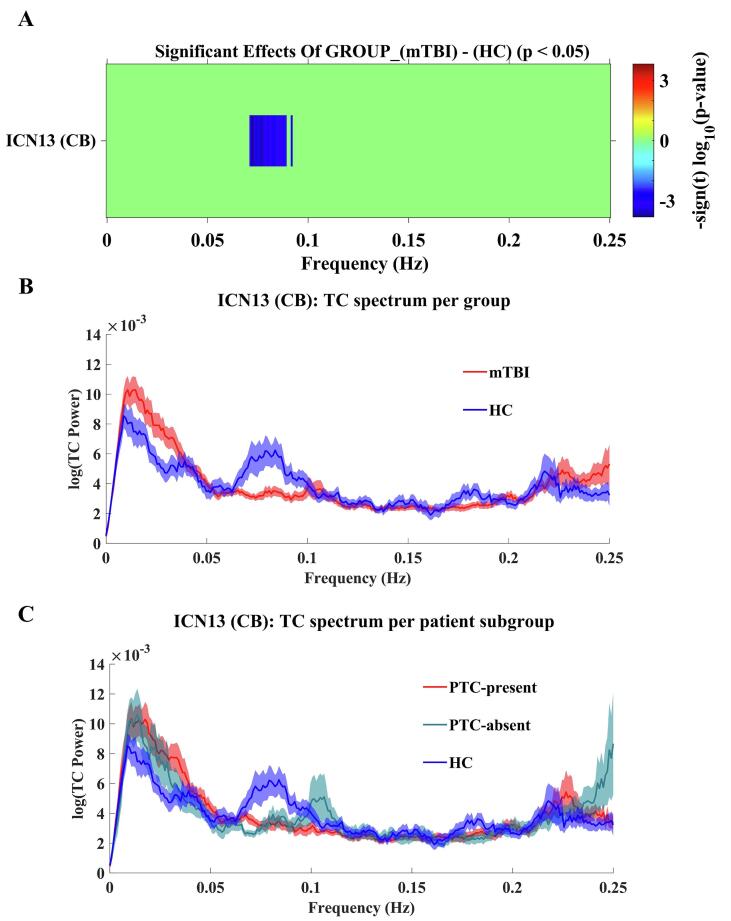
Effects of Group (mTBI vs. HC). (A): the color bar displays the significance and direction of the effects of group for ICN13 (cerebellar network) in the TC power spectrum (p < 0.05, FDR-corrected). (B): Line plots of the average TC power spectra for mTBI patients (mTBI; red) and healthy controls (HC; blue). C): Line plots of the average TC power spectra for mTBI patients with PTC-present (PTC-present; red), mTBI patients with PTC-abstent (PTC-absent; green) and healthy controls (HC; blue). The line plots show mean log(power) ± 1SE. (For interpretation of the references to color in this figure legend, the reader is referred to the web version of this article.)

For low frequencies, within the 0.065–0.10 Hz range, TC power was significantly lower for mTBI patients in comparison to HC for ICN13 (CB; p < 0.05, FDR-corrected), suggesting abnormal deactivation in the cerebellum after mTBI. These results are illustrated in Fig. 6A and B. Additionally, the average TC spectra for ICN13 per subgroup of mTBI patients are detailed in Fig. 6C. The results of univariate tests on TC power spectra per patient subgroup (PTC-present and PTC-absent) vs. HCs can be found in the in the Supplementary Material (Section C2, Fig. S3).

## 4 Discussion

In this study, we used group ICA to identify fMRI-ICNs in a sample of mTBI patients and HCs aged 18–64 years. We aimed to identify how age and mTBI affect brain connectivity and whether their effects can be separated. We employed a multivariate analysis to identify the effects of age and mTBI on between- and within-network connectivity while controlling for sex and head motion. To investigate differences between the two patient subgroups (PTC-present and PTC-absent) and to include the interval between the accident and the scan (in days) as well as the GCS score as nuisance covariates, we built a second model (see Supplementary Material, sections A and E).

Consistent with previous studies, we identified age-related changes for all of the three connectivity measures that were analyzed (Allen et al., 2011, Geerligs et al., 2015). Although the ventral medial prefrontal cortex (vmPFC) area, represented by ICN23 (anterior DM), was most profoundly affected by age, presenting age-related changes for both between-network connectivity (FNC) and within-network connectivity (TC power spectra) measures, our findings revealed significant age-related changes for connectivity between components (FNC) across all ICNs, suggesting global changes in the brain connectivity architecture. We consistently observed a linear correlation between the FNC measures and the logarithmic transformation of age, indicating that more pronounced changes occur during young adulthood and progress at a slower pace during mid- to late-adulthood.

Furthermore, we found significant changes related to age for within-component connectivity for both outcome measures (SMs and TC power spectra). Both age-related decrease and increase for SM intensity were identified in clusters located in subcortical and cognitive control areas. Additionally, an age-related decrease for SM intensity was identified in clusters located in sensorimotor and visual areas. Age-related changes for TC spectra were identified in three ICNs (one from the CC, two from the DM) located in frontal brain regions. For all three frontally located ICNs we identified a power decrease for lower frequencies (below 0.15 Hz) and a power increase for higher frequencies (above 0.15 Hz) suggesting that a single process might be underlying this apparently local trend.

No significant changes related to mTBI (compared to HC) were found for connectivity between components (FNC) and significant changes related to mTBI for within-component connectivity (SM intensity and TC power spectra) were found only for the TC spectra of the cerebellar ICN. It suggests that effects of mTBI for static functional connectivity between components are absent or much subtler than those of age, but that local changes in coherent activity within ICNs might occur after mTBI. Moreover, the interaction group by age was never retained in the multivariate analysis, suggesting that mTBI does not affect an older brain more (nor less) than an younger brain for the age range and connectivity measures investigated in this study.

The findings of the second model were consistent with and complimentary to those of the main model. In the multivariate analysis of the second model, similar to the first model, age was retained as a significant predictor for all of the three connectivity measures and the only significant predictor for FNC. The results also support the incorporation of sex and FD as nuisance covariates for measures of connectivity within networks. Additionally, the terms subgroup and the interaction subgroup × age were found to be weak significant predictors for TC spectra of several ICNs. Surprisingly, the two additional nuisance covariates (GCS score and time interval between accident and scan) were retained as weak significant predictors for TC spectra and SMs of few ICNs, suggesting that their effects on brain connectivity are lower than the effects that are associated with the other covariates. When comparing the results of the univariate analysis between both models, similar effects for age were found for all of the three measures (FNC, SMs and TCs). Small differences (i.e. different p-values) were found, as expected due to a smaller number of participants in the second model (mTBI patients only, N = 54) compared to the main model (mTBI patients and HCs, N = 74). A few significant effects for subgroup (PTC-present vs. PTC-absent) were found for the TCs of two ICNs (ICN 27 (CC) and ICN 17 (VIS)). Significant effects for the interaction term subgroup × age were found for SM intensities of ICN 19 (SMO) only. In spite of this interesting finding, the cluster size was small (V_l_ = 2) and results should be reproduced in future studies before further interpretation.

At last, the point in time of scanning is an important matter for mTBI research. In our study, we opted to scan at four weeks after injury as one of the main goals of the study was to examine alterations of brain connectivity associated with the increased risk of developing persistent symptoms. It could be argued that, after four weeks, potentially pathological effects of mTBI in resting-state brain connectivity might have been mitigated. However, several cross-sectional and longitudinal mTBI studies have shown altered brain connectivity at four weeks post-injury or beyond (see van der Horn et al., 2016a, van der Horn et al., 2016b for a review). For example, Mayer et al. (2011) identified altered connectivity at two weeks post-injury that persisted for (at least) four months in comparison to HCs. Iraji et al. (2016) found similar levels of hyperconnectvity in mTBI patients at the first day and at four to six weeks after mTBI. A more recent study by Meier et al. (2017) found that differences in functional connectivity were stronger at one month than at one or at two weeks after injury, in comparison to HCs. Unfortunately, none of these three studies added age as a covariate and not all of them age-matched groups. Therefore, direct comparison with our results is not possible.

In the following subsections, we will discuss changes in between- and within-network connectivity, separately, and will review our findings in relation to previous mTBI studies.

### 4.1 Between-network connectivity: FNC – Age-related changes and mTBI

Our results match and extend the findings from previous studies (Sours et al., 2015, van der Horn et al., 2016a, van der Horn et al., 2016b), where the FNC between none of the component pairs was significantly different between the mTBI patients and HCs based on cross-sectional analyses with data acquired during the subacute phase.

Previous research on mTBI patients indicates that interactions between the salience network (SN) and the DM can be disrupted after brain injury, possibly leading to impairments in cognitive control (Sharp et al., 2014). The SN is involved in processing of external salient stimuli and is part of the CC (domain), with key areas located in the anterior cingulate cortex (ACC) and in the anterior insula (AI). In this study, the SN is captured by ICNs 15, 16 and 18 (CC). Here, we did not find any changes for between-network connectivity involving ICN pairs that correspond to the DM and the SN. Perhaps changes would be more likely when administering a cognitively challenging task (e.g. go-no go task), where processing of external salient stimuli is actively required (Bonnelle et al., 2012).

Previous studies also indicated that higher functional connectivity between the anterior and posterior components of the DM in the early subacute phase was associated with a greater number of PTCs in the late subacute phase (Van Der Horn et al., 2017). These PTCs include complaints related to emotional, cognitive and/or physical functioning. In this study, the anterior and posterior components of the DM are represented by ICNs 23 and 11, respectively. Although we did not identify changes for FNC between the DM pair ICN 23 and ICN11, all of the measurements of our study were performed (cross-sectionally) in the early subacute phase and using a multivariate approach in an exploratory fashion, while the aforementioned study investigated how connectivity changes in pre-defined ICNs correlate with PTCs longitudinally in age-matched groups, without adding age as a covariate. Therefore, a direct comparison of results is not possible.

With respect to age-related changes in FNC, our results indicate that the anterior DM (ICN23) and CC (ICN16 and ICN18) are more strongly affected by aging: nine out of the 10 pairs involved ICN from the DM (most frequently ICN23; anterior DM) and/or an ICN from the CC (most frequently ICN16 or ICN18). The remaining pair corresponds to ICN13 (CB) and ICN17 (VIS). In the next paragraphs, we will discuss the most relevant findings for age-related changes in our study, with focus on pairs involving ICNs of the DM and of the CC domains. An extension of the discussion can be found in the Supplementary Material (section E).

In this study, we found increased as well as decreased correlation between FNC and age across ICN pairs. This is consistent with the notion that brain connectivity between ICNs changes throughout adulthood with increasing age, but neither decreasing nor increasing ICN connectivity with aging can be taken as a general rule (Geerligs et al., 2015). Importantly, increased correlation can also reflect a reduction in anti-correlation, which means that interpretation is not straightforward.

The presence of anticorrelations between ICNs of the DM and ICNs from other domains has been observed and explored in previous studies (Fox et al., 2005, Uddin et al., 2009) and usually pertains to connections between the DM and the so-called ‘task-positive’ network (TPN; Fox et al., 2005). Correlation increase is mainly associated with neural dedifferentiation. According to the dedifferentiation theory, functional areas of the brain become less distinct (increasingly correlated) in elderly (Baltes and Lindenberger, 1997, Park et al., 2004). This concept has been extended to functional networks (Chan et al., 2014, Geerligs et al., 2014, Koen et al., 2020). It is worth noting that although dedifferentiation is generally associated with age-related performance loss, less distinctive functional networks could also reflect a compensatory mechanism (Cabeza, 2002). Particularly, increased connectivity involving frontal areas (e.g. PFC), which are associated with high cognitive processing, seems to reflect an age-related compensation phenomenon that was termed ‘Posterior to Anterior Shift in Ageing’ (PASA; Davis et al., 2008). Dedifferentiation and compensation are unlikely to be mutually exclusive (Burianová et al., 2013, Geerligs et al., 2015). Because the current study used resting-state fMRI, in contrast to task-based fMRI, it is not possible to provide direct evidence of which mechanism (dedifferentiation and/or compensation) plays a stronger role in our sample population regarding changes in connectivity.

In our study, we found a decrease in anticorrelation between ICN23 (DM) and ICN27 (CC) slowly progressing with log(age). ICN27 (CC) includes the bilateral dorsolateral prefrontal cortex (dlPFC) which is activated during cognitive tasks involving working memory (WM) and attention (Barbey et al., 2013, Petrides, 2000). Additionally, we observed an increase in correlation between ICN23 (DM) and ICN16 and ICN18. Both ICN16 and ICN18 are part of the cognitive domain and are associated with language functioning. It could be argued that these findings indirectly reflect the cognitive deficits and compensation mechanisms that are expected with aging, indicating that this process starts at a younger age.

In addition to the possibility that an mTBI does not significantly affect FNC (at rest), we can speculate that the effects of aging are more pronounced than those resulting from an mTBI for the lifespan of the population in this study (18–64 years old) and effects of mTBI on FNC might be reduced after the acute phase.

### 4.2 Within-network connectivity – age-related changes and mTBI

The SM together with BOLD TC spectra are the main features of an ICN. Changes in SM intensities can indicate abnormalities in co-activation patterns of voxels within networks. Changes in BOLD spectral power can indicate abnormalities in coherent activity patterns within the global time course of networks.

#### 4.2.1 Spatial map intensities

Previous fMRI studies on mTBI patients found both increases and decreases in within-network connectivity in ICNs of the DM, SN (CC) and ICNs of other domains, reporting that these abnormalities correlate with cognitive deficits or post-concussive complaints (Messé et al., 2013, Sharp et al., 2014, Stevens et al., 2012). During task performance, increased activation is also frequently observed in ICNs involved in cognitive control and without deficits in performance, suggesting a compensatory mechanism. Reduced activation has been reported in the ICNs of the SMO, involved in motor tasks.

In this study, we did not find any significant changes in ICN SM intensities when comparing mTBI patients in the subacute phase post-injury to HCs or when comparing mTBI patient subgroups (PTC-present to PTC-absent). However, we did find age-related increased intensities in the ICN15 (CC; partly contains the SN) and decreased intensities in the SMO both in the main model including mTBI patients and HCs and in a second model including only mTBI patients (subgrouped by presence or absence of complaints).

This suggests that age affects SM intensity patterns in cognitive and motor related ICNs, among others. Additionally, effects of aging in SM intensities develop slowly with time and can be robustly identified when analyzing a broad lifespan compared to effects of mTBI, which might be more difficult to measure (or absent).

#### 4.2.2 TC power spectra

We found reduced coherent cerebellar activity for mTBI patients within the range of the BOLD TC spectra where neural activation is more reliably captured. Our results indicate power decreases for frequencies between 0.065 and 0.10 Hz in the TC spectra of ICN13 (cerebellar network) in mTBI patients relative to HCs. A closer inspection of the average TC power spectra from both groups actually indicated an absent “power peak” between 0.065 and 0.10 Hz for mTBI patients. When further inspecting the average TC power spectra from mTBI subgroups in an explorative manner, we observed a late, lower “power peak” between 0.10 and 0.11 Hz for mTBI PTC-absent patients, in contrast to no ‘peak’ for the mTBI PTC-present group. These results suggest that abnormal deactivation in the cerebellum after mTBI might be associated with the presence of complaints.

Our results are in line with previous studies that found regional reduced activity in the cerebellum for mTBI patients. Previously, Peskind et al. (2011) found that cerebellar glucose hypometabolism in war veterans after blast-induced mTBI was associated with subtle impairments in aspects of cognitive processing speed, attention and working memory. Additionally, Stevens et al. (2012) found that functional connectivity within the right cerebellum posterior lobe (among other areas) negatively correlated with post-concussive symptoms. Furthermore, Zhan et al. (2016) reported reduced power between 0.01 and 0.08 Hz in the left posterior lobe of the cerebellum for a group of acute mTBI patients at rest. All these previous results suggest that the cerebellum is a relevant part in the mechanisms involved in the development of cognitive symptoms after mTBI. These results may be explained by the fact that direct and indirect damage to the cerebellum might occur after mTBI (Potts et al., 2009, Vergara et al., 2018).

Anatomically, the cerebellum is connected to the PFC through polysynaptic projections via the thalamus. Functionally, the cerebellum has a traditionally recognized role in motor control. There is increasing evidence that the cerebellum is also involved in cognitive and emotional control, but the detailed characterization of its functional role is still unclear. Recent studies based on functional connectivity suggest a homotopic representation of the cerebellum that is coupled to the cerebral cortex (Buckner, 2013, Buckner et al., 2011, Schmahmann, 2019). Furthermore, it has been demonstrated that transcranial magnetic stimulation (TMS) applied to specific areas in the cerebellum can modulate the activity within the (cerebral) DM and other networks.

Clinical studies also suggest that, in addition to ataxia and impaired fine motor control, cognitive and affective deficits might occur after cerebellar damage (Alexander et al., 2012, Gottwald et al., 2004, Ravizza et al., 2006). The latter however, are subtle and transient in comparison to the more obvious motor deficits.

With respect to age-related changes in TC power spectra, we consistently found significant spectral power decreases for low BOLD frequencies (below 0.15 Hz) and significant spectral power increases for high BOLD frequencies (above 0.15 Hz) for three ICNs mainly located in the medial PFC: for ICN23 (anterior DM), including the vmPFC; for ICN15 (CC), including the dorsomedial prefrontal cortex (dmPFC) and for ICN5 (DM), including the orbitofrontal prefrontal cortex. Interestingly, all three components are medially located in the frontal lobe and share a similar trend of changes in (intra-component) coherent activity. These results suggest common underlying mechanisms that are either restricted to or intensified at areas involved in higher order cognitive processes and that also present a similar pattern for changes in the power spectra of TCs with age as previously identified by Allen et al. (2011) (on a broad population of HCs). Previous studies associated power decreases in frontal areas at low frequencies (below 0.1 Hz) to deterioration of cognitive performance (Fryer et al., 2015, He et al., 2013). Other studies have suggested that higher frequency BOLD oscillations (>0.1 Hz) are associated with non-neural fluctuations (e.g. vascular artifacts, respiratory rhythms) (Birn et al., 2006, Wise et al., 2004). However, there is increasing evidence that higher frequency BOLD oscillations (>0.1 Hz) also reflect neural activity (Chen and Glover, 2015, Gohel and Biswal, 2015, Jahanian et al., 2019) and that increased power in higher frequency oscillations may be indicative of reduced connectivity within the ICN (Balsters et al., 2013a). Our findings also match other studies that kept frequencies between 0.1 and 0.25 Hz in the analysis and found increasing high frequency power with age (above 0.2 Hz) for several ICNs (Allen et al., 2011, Balsters et al., 2013a).

Our results revealed decreasing power for increasing age at the lower end of the spectra, mainly below 0.1 Hz, where intrinsic fluctuations resulting from activations in gray matter can be typically identified. Our findings are in line with a number of other studies reporting that within-network connectivity in frontal areas in the brain (including the DMN) decreases with age (Achard et al., 2006, Glerean et al., 2012). Similar to the findings of (Geerligs et al., 2015), our observed significant power decrease within ICNs was restricted to high-cognitively demanding areas. Interestingly, Allen et al. (2011) investigated a broad population of HCs and found decreasing low frequency power with age (below 0.15 Hz) for all 28 ICNs they identified and stronger age-related effects were found in fronto-parietal (CC) and DM ICNs. Although there are some methodological differences with our study (e.g. Allen et al. (2011) investigated a relatively younger, larger population and acquired data during resting state with eyes-open), the results are not conflicting. Altogether, their and our results suggest an underlying global age-related trend in within-network connectivity changes that locally affect the DM and the frontal/fronto-parietal areas more strongly and may be associated with cognitive decline. Lastly, a closer inspection of the average TC spectra per age group (median split), revealed decreased low-frequency power in ICN15 (CC) for older participants. The “deactivation” of components in the cognitive domain ICN15 (CC) together with decreased (anti-) correlation between components in frontal areas (see 4.1) indicates decreased segregation and reduced activation of networks in the frontal lobe for increasing age, in line with the previously discussed dedifferentiation hypothesis.

In the aging brain, we could speculate that abnormal cerebellar activity acts as a stressor in an already adapted, cognitively vulnerable brain organization, facilitating the development of PTCs. Because altered activity in the cerebellum can modulate the activity in networks that are located in the PFC, this could also facilitate well established effects of age on brain connectivity within frontally located networks and between components pairs involving the anterior DM.

It is therefore tempting, although very speculative, to suggest that abnormal activity of the cerebellum in mTBI contributes to the development of PTCs. Lastly, how neural processes in the cerebellar structure can lead to changes in the BOLD signal also remains under investigation and, therefore, suggesting that abnormal activity in the cerebellum leads to PTCs would be based on indirect evidence.

## 5 Limitations and future directions

In this study, children (below 18 years old) or elderly participants (above 65 years old) were not included. Therefore, considering the age of the population investigated in this study (median age: 32 years (IQR: 23–51), range 18–64 years), it would not be advisable to extrapolate its results to very young nor to elderly population. Additionally, protective effects related to cognitive reserve, for example, might play a stronger role in older age. Thus, it would be interesting for future studies to investigate brain network connectivity involving the elderly population including covariates related to protective factors.

Moreover, the cerebellum has been omitted or received limited attention in several studies analyzing functional connectivity, given that the main focus has traditionally been on the cerebral cortex, especially on the DMN. Additionally, while several studies on functional connectivity analyze correlation between BOLD TCs and/or alterations in the intensity of ICN spatial maps, underlying changes in the TC spectra are rarely made explicit. We therefore hope that future studies on connectivity will explore the temporal and spectral characteristics of all ICNs, including (but not limited to) the DMN.

With regard to the effects of mTBI, in addition to the presence/absence of PTCs, how different complaints relate to changes in functional connectivity remains a matter for further investigation. It is also known that mTBI patients compose a heterogeneous group where, in addition to age, sex and education level, other factors as cognitive reserve and pre-injury mental state might play a role in brain functioning. Further research investigating the effects of additional demographic and clinical factors, if possible longitudinally, is thus encouraged.

Furthermore, in the current study we opted to scan at four weeks after injury to assess changes that are associated with mTBI-related pathology and long-lasting symptoms. However, the optimal point in time for this purpose remains unknown. Perhaps we could have been more sensitive at an earlier point in time. We encourage future longitudinal mTBI studies that help elucidating the course of mTBI pathology.

Finally, the source and the functional meaning of the BOLD low-frequency oscillations (LFOs) remains under investigation (Klimesch, 2018). There is growing evidence that features of LFOs are related with cognitive abilities and behavior (Balsters et al., 2013b, Fryer et al., 2015, Mennes et al., 2011). In parallel, speculative conclusions that BOLD and electrophysiological signals share the same underlying phenomenon are accumulating (Balsters et al., 2013a, He et al., 2008, Palva and Palva, 2012). Cross-modal studies (e.g. combined EEG and fMRI) are gaining momentum and might help elucidate how changes in the BOLD spectra relate to cognition and behavioral aspects in health and disease (Abreu et al., 2020, Sadaghiani and Wirsich, 2020).

## 6 Conclusion

This study demonstrates that effects of mTBI on between-network functional connectivity, if any, are much subtler than those of aging and reinforces the importance of adding age as a covariate in mTBI studies, in addition to age-matching groups. Aging, regardless of mTBI, strongly affects functional connectivity over the whole brain, with effects for between-network connectivity being most evident in pairs involving the anterior DMN and/or the CC; for within-network connectivity being most evident in frontal areas. MTBI effects can be detected as abnormal coherent activity in the cerebellum within a specific low-frequency range (0.065–0.10 Hz) and seem to be associated with presence of post-traumatic complaints. Future cross-modal functional connectivity studies investigating the interaction between cerebellar and DM ICNs and evidencing spectral properties of the BOLD signal are encouraged.

## 7 Declarations of interest

None.

## CRediT authorship contribution statement

**M. Bittencourt-Villalpando:** Conceptualization, Methodology, Software, Formal analysis, Writing - original draft. **H.J. van der Horn:** Conceptualization, Methodology, Investigation. **N.M. Maurits:** Supervision, Writing - review & editing. **J. van der Naalt:** Supervision, Writing - review & editing.

## Footnotes

1 http://mialab.mrn.org

2 http://www.cis.hut.fi/projects/ica/icasso.

3 http://www.mathworks.com/matlabcentral/fileexchange/27014-mancovan.

4 http://afni.nimh.nih.gov/afni.

5 http://chronux.org.
