# Supplementary material for: Disentangling the effects of age and mild traumatic brain injury on brain network connectivity: A resting state fMRI study
Source: Neuroimage Clin. 2020 Dec 22;29:102534. doi: 10.1016/j.nicl.2020.102534 (PMC7770973; doi:10.1016/j.nicl.2020.102534)
Supplement: Supplementary data 1 [file mmc1.docx]

**SUPPLEMENTARY MATERIAL**

**Disentangling the effects of age and mild traumatic brain injury on brain network connectivity: A resting state fMRI study**

Bittencourt-Villalpando, M.; van der Horn, H.J.; Maurits, N.M.; van der Naalt, J.

Department of Neurology, University Medical Center Groningen

**A. Methods – Additional statistical analysis: second model**

For the second model (B: PTC-present vs PTC-absent), the design matrix included three covariates of interest: subgroup as a categorical variable (PTC-present or PTC-absent), age as a continuous variable and the interaction term group by age. In addition, we included four nuisance covariates: sex and FD as previously described for model A; time interval between accident and scan [Tscan] as a categorical variable (0: Tscan ≥ 33 days; 1: Tscan < 33 days) and GCS (0: GCS = 15; 1: GCS < 15).

**B. Methods – Additional information on MANCOVAN toolbox**

FNC values were extracted from the gig_mancovan_results_fnc.mat output matrix and inverse Fisher-transformed.

SM z-score values were extracted from the gig_mancovan_results_sm_xxx.mat output matrix, where xxx is the ICN number.

TC power spectra values were extracted from the gig_mancovan_results_spectra_xxx.mat output matrix, where xxx is the ICN number.

**C. Supplementary Results – Main model**

C1. FNC

The scatterplots in Fig. S1 depict the results of the univariate tests for effects of age on FNC between components that are presented in Section 3.3.1 (Fig. 3B) for the remaining eight ICN pairs that were not depicted in the main document as the examples in Section 3.3.1 (Fig. 3C, D).


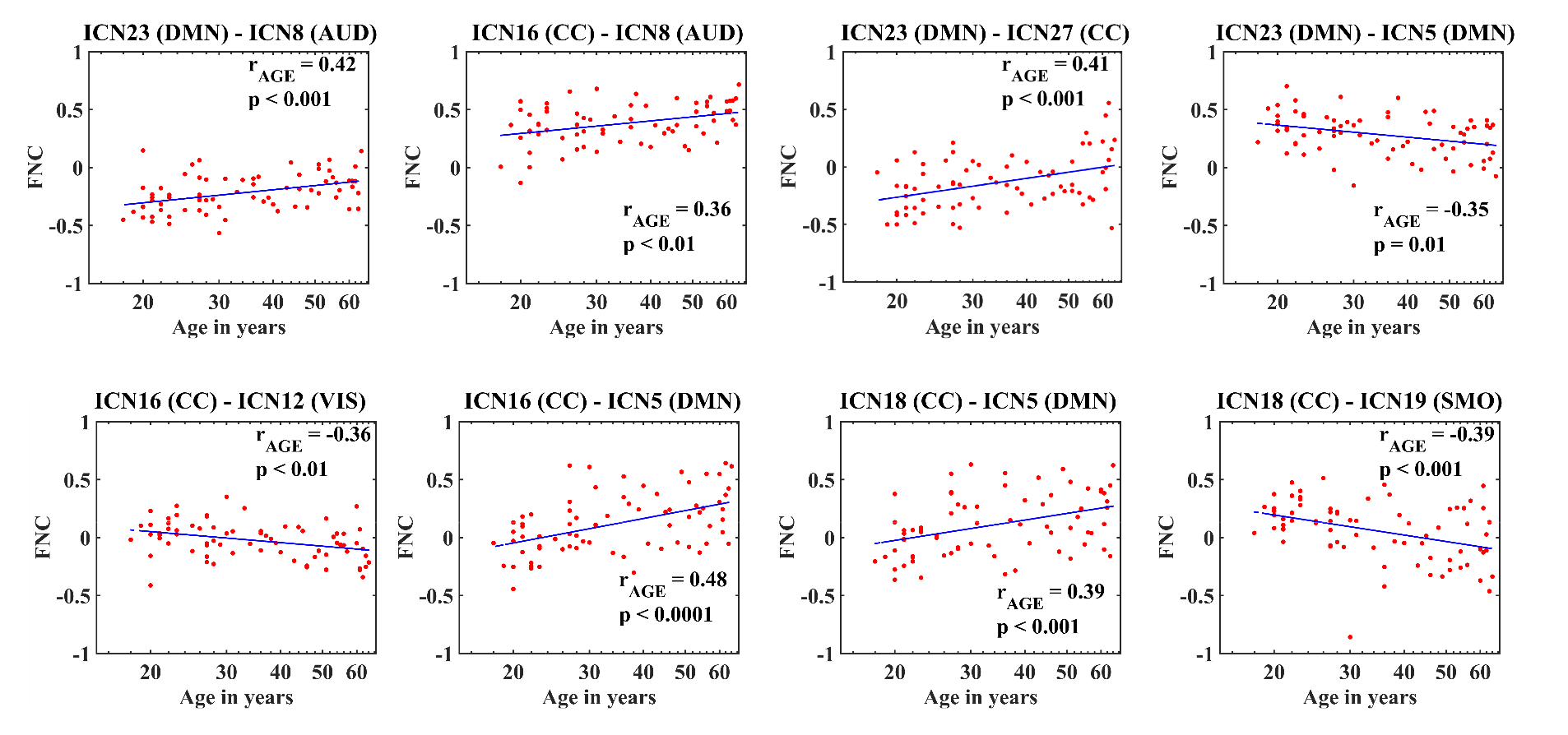


Figure S1- Effects of age on (static) functional network connectivity (FNC) between components for the eight out of 10 ICN pairs in Table 3 that were not used as an example in Fig. 3. Scatterplots depict significant changes in FNC for increasing log(age).

C2. Timecourse spectra

The scatterplots in Fig. S2 depict the results of the univariate tests that are presented in Section 3.3.3 (Fig. 5A). The scatterplots in Fig. S2A and Fig. S2B show the results for ICN15 (CC), where we found a negative correlation between log(Age) and average log(power) for low frequencies (r_AGE_ = -0.46, p<0.0001) and a positive correlation between log(Age) and average log(power) for high frequencies (r_AGE_ = 0.43, p<0.001). Similarly, the scatterplots in Fig. S2C and S2D show the results for ICN5 (DMN), where we found a negative correlation between log(Age) and average log(TC power) for low frequencies (r_AGE_ = -0.37, p<0.01) and a positive correlation between log(Age) and average log(TC power) for high frequencies (r_AGE_ = 0.41, p<0.001).


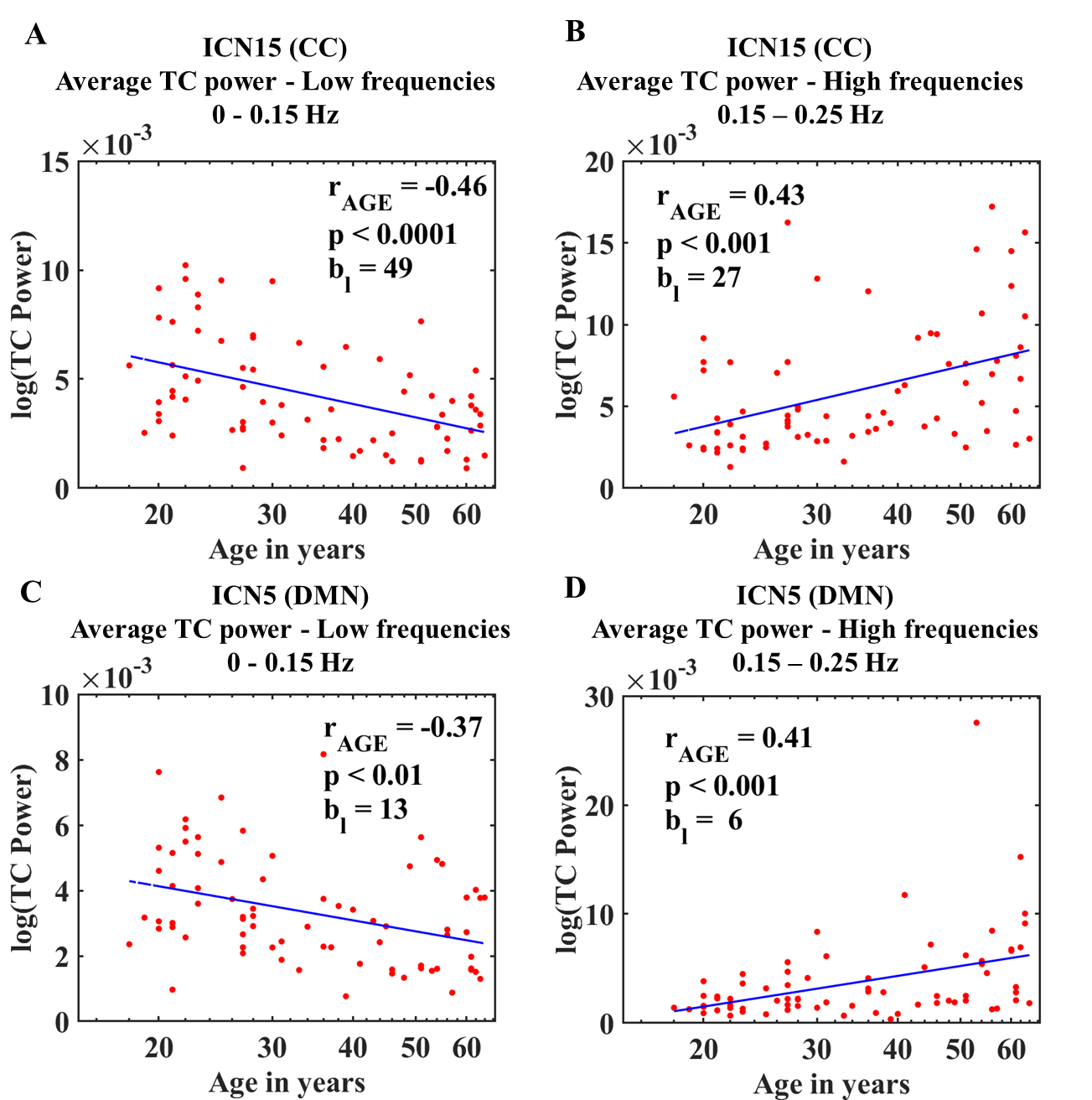


Figure S2 – Effects of age on TC power spectra. (A, B): Scatterplots depicting changes in TC power for increasing age for ICN15 (CC). (C, D): Scatterplots depicting changes in TC power for increasing log(age) for ICN5 (DM).

Fig. S3 shows the results of the univariate tests on TC spectral power for the subgroup mTBI PTC-present vs. HCs. For low frequencies, within the 0.065-0.10Hz range, TC power was significantly lower for mTBI PTC-present patients in comparison to HCs for IC13 (CB; p<0.05, FDR-corrected), suggesting abnormal deactivation in the cerebellum after mTBI in patients with PTCs. No significant effects were found after univariate tests on TC spectral power between the subgroup mTBI PTC-absent vs. HC nor between the subgroup mTBI PTC-present vs. the mTBI PTC-absent group.


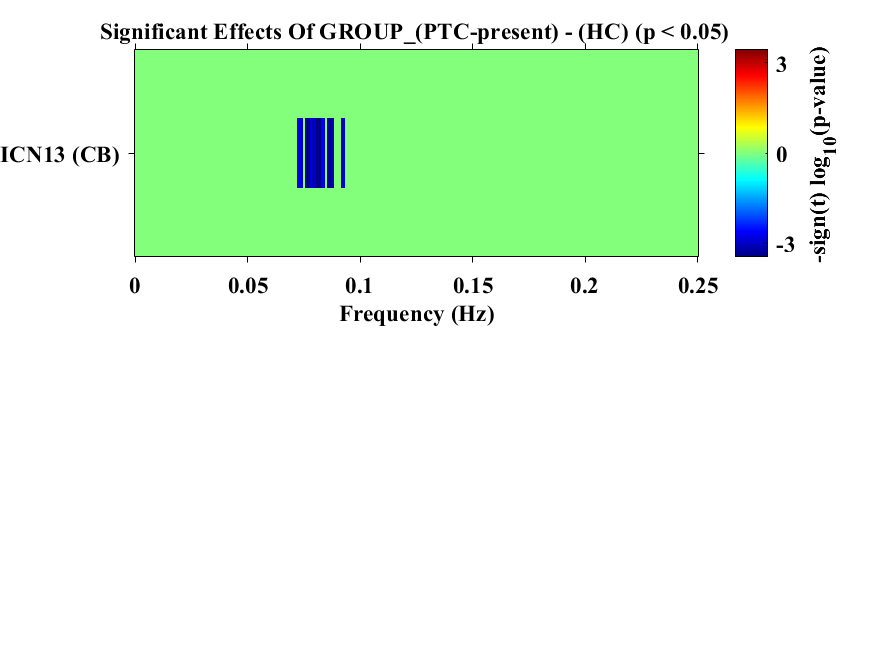


Figure S3 - Effects of subgroup (mTBI PTC-present vs. HC): the color bar displays the significance and direction of the effects of group for the ICN13 (cerebellar domain) in the TC power spectrum (p<0.05, FDR-corrected).

**D. Supplementary Results – Second model**

D1. Multivariate results


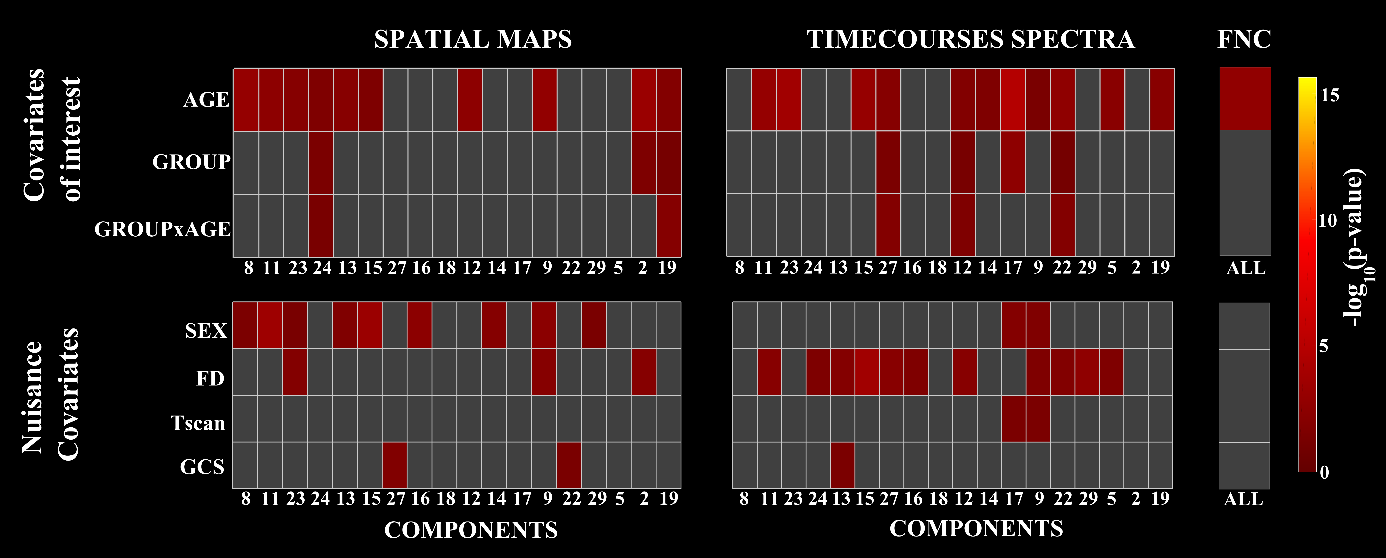


**Figure S4 - Results from the multivariate tests showing the significance of the covariates of interest and nuisance predictors for Spatial Map intensities, TC power spectra and FNC.** Gray squares represent model terms that were not retained in the backward selection process (α = 0.05).

D2. FNC

Fig. S5 illustrates the effects of age on FNC between ICNs for the second model (mTBI patients only). These significant results were found in five ICN pairs that were also among the 10 ICN pairs identified in the main model but with lower p-values as expected due to a smaller number of participants in the second model (mTBI patients only, N=54) than in the main model (mTBI patients and HCs, N=74). Therefore, results were not further depicted in scatterplots (because similar results would be expected).


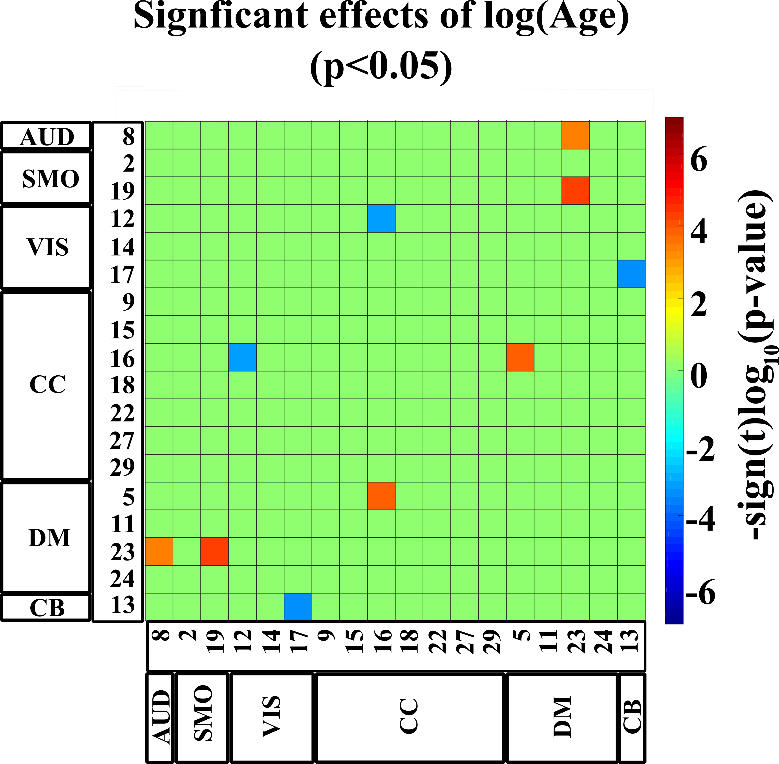


Figure S5 - Effects of age on (static) functional network connectivity (FNC) between intrinsic connectivity networks (ICNs). The matrix displays the significance and direction of the effects of age for each pairwise correlation (p<0.05, FDR-corrected).

D3. Spatial Maps

The effects of age on SM intensities for the second model (mTBI patients only) are shown in Fig. S6. Significant effects were found in ICN2 (SMO) and ICN15 (CC).


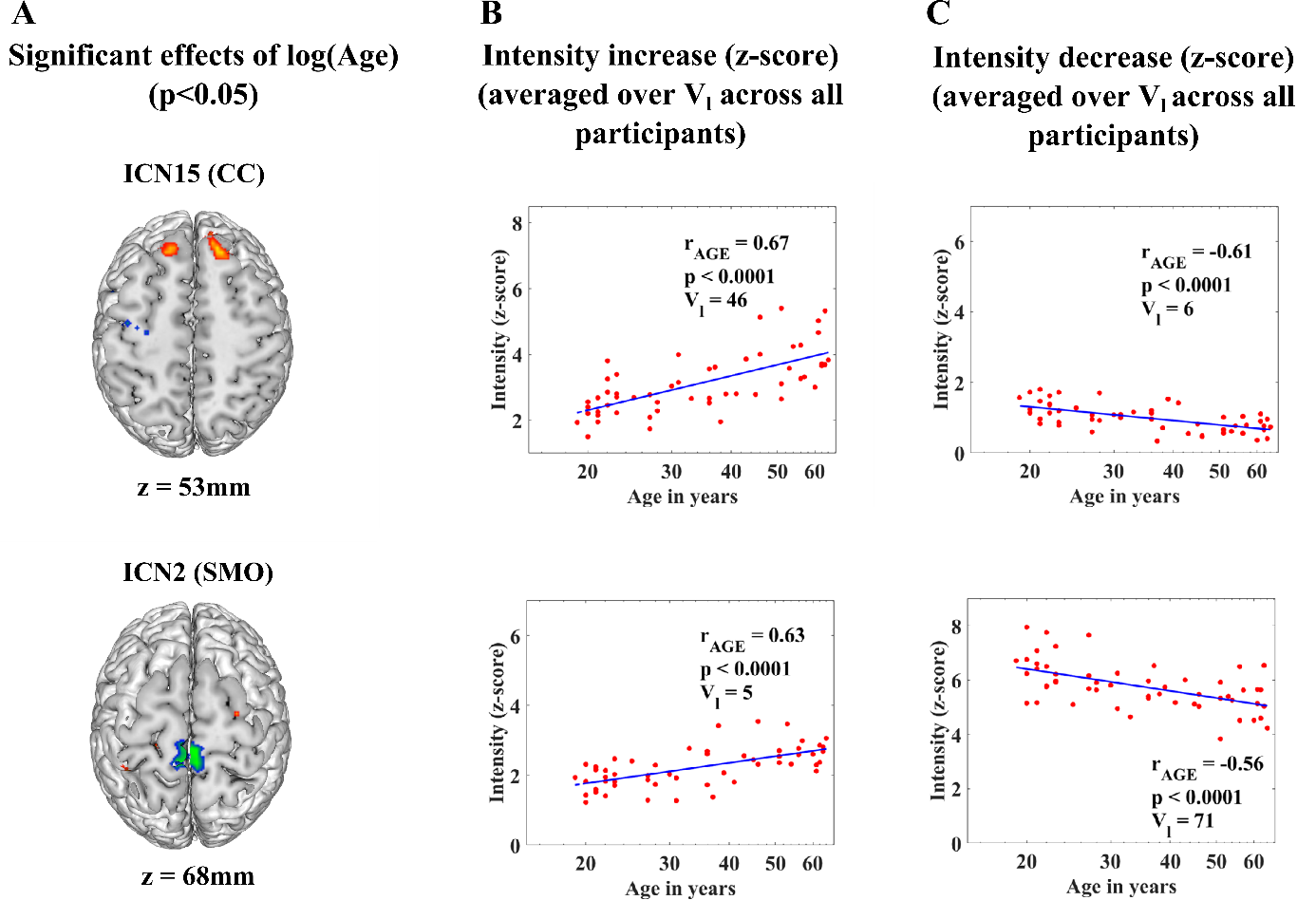


Figure S6 - Effects of age on SM intensities of mTBI patients. (A): Significant effects of age for each ICN SM (p<0.05, FDR-corrected). (B;C): Scatterplots depicting the significant changes in SM intensities for log(age). Age is presented in the scatterplots (B) and (C) on a log-scale.

The effects of the interaction age×subgroup on SM intensities for the second model (mTBI patients only) are shown in Fig. S7. Although the effects found for the SM intensities of ICN19 (SMO) are significant, the cluster size is small (V_l_=2) and results should be interpreted with caution.


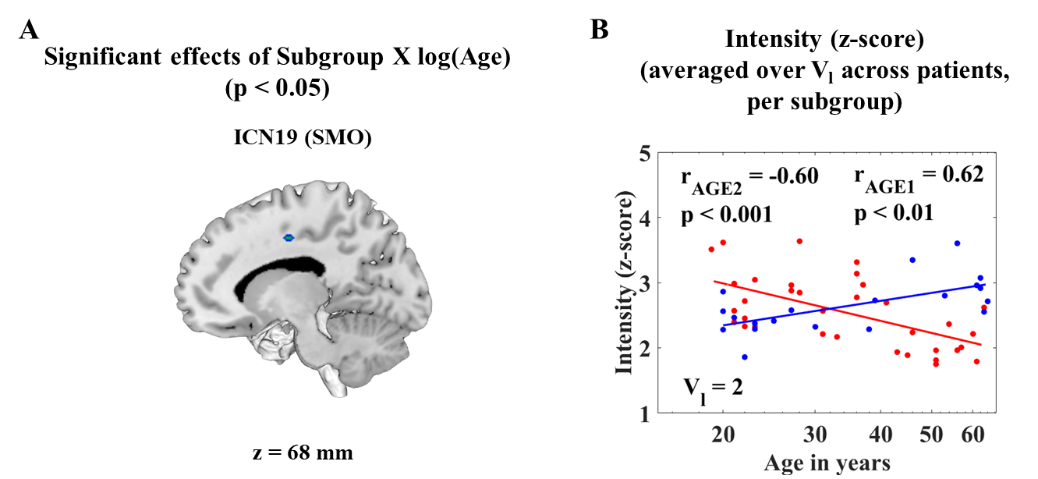


Figure S7 – Effects of age×subgroup interaction on SM intensities of mTBI patients. (A): Voxels with significant age×subgroup interaction effect for ICN SM intensity (p<0.05, FDR-corrected). (B): Scatterplot depicting the significant results of changes in SM intensities. Colors indicate patient subgroups (blue: PTC-absent; red: PTC-present). Age is presented in the scatterplot (B) on a log-scale.

D4. Timecourse spectra

For low frequencies, below 0.15 Hz, TC power decreased significantly with age in mTBI patients, for ICNs 5, 15 and 23 (p<0.05, FDR-corrected). For these same ICNs, TC power increased significantly with age for higher frequencies, above 0.15 Hz (p<0.05, FDR-corrected). These results are illustrated in Fig. S8 and are similar to the results found for the main model (mTBI patients and HCs). Therefore, results were not further depicted in scatterplots (because similar results would be expected).


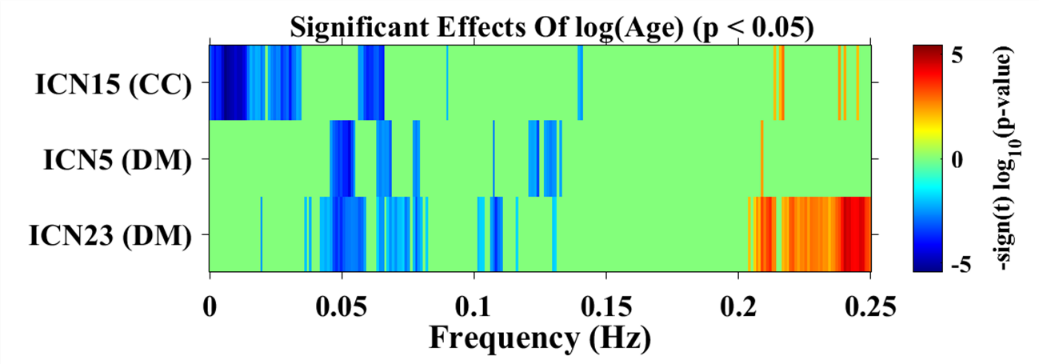


Figure S8 - Effects of age on TC power spectra of mTBI patients. (A): Effects of age for each IC TC power spectrum; the color bars display their significance and direction (p<0.05, FDR-corrected).

For low frequencies, within the 0.05-0.08Hz range, TC power was significantly higher for mTBI patients with PTC-present in comparison to mTBI patients with PTC-absent for ICN17 (VIS; p<0.05, FDR-corrected) and for ICN27 (CC; p<0.05, FDR-corrected), suggesting either abnormal activation in some regions of the visual and cognitive domains after mTBI in patients with PTC-present or abnormal deactivation in some regions of the visual and cognitive domains after mTBI in patients with PTC-absent. These results are illustrated in Fig. S9.


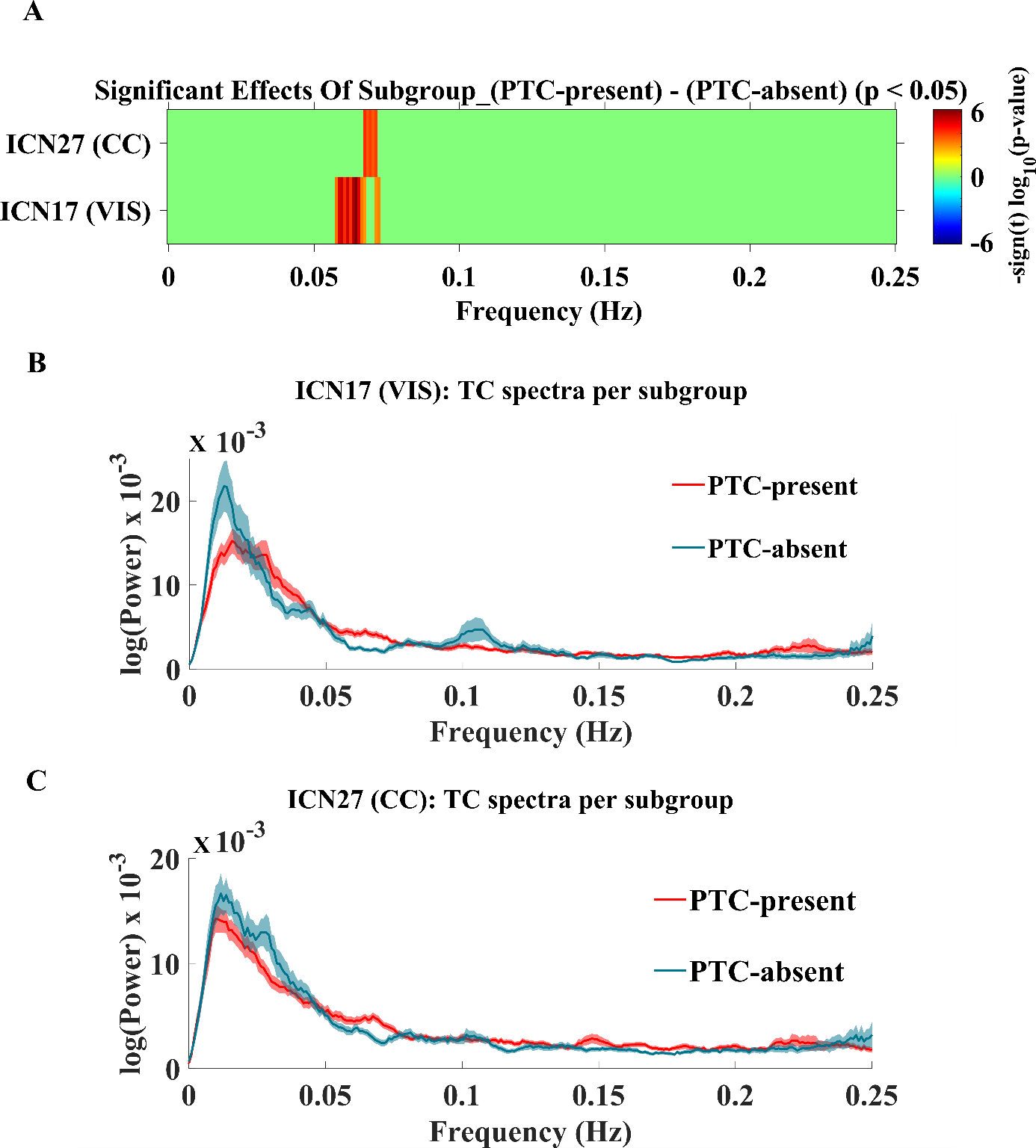


Figure S9 - Effects of patient subgroup (mTBI PTC-present vs. mTBI PTC-absent). (A): the color bar displays the significance and direction of the effects of group for ICN17 (VIS) and ICN27 (CC) in the TC power spectrum (p<0.05, FDR-corrected). (B, C): Line plots of the average TC power spectra for mTBI patients with PTC-present (PTC-present; red), mTBI patients with PTC-absent (PTC-absent; green). The line plots show mean log(power) ±1SE.

**E. Supplementary Discussion: Age-related FNC changes**

In addition to the age-related changes in FNC discussed in the main file, we found a combination of effects (decrease in anticorrelation from young age up to mid-adulthood followed by increase in correlation from mid-adulthood to older age) for the pair ICN19 (SMO) and ICN23 (DM). Our findings possibly reflect a combination of two progressive age-related effects: loss of motor control skills and compensation mechanisms that emerge, the latter in line with the PASA theory (Davis et al., 2008).

We also observed a decrease in anticorrelation between ICN8 (AUD) and ICN23 (DM) with age. We could speculate that our findings reflect age-related deficits in auditory functions that might be explained by reduced ability to direct attention to external auditory stimuli. Alternatively, these findings might indicate increased internal processing of auditory information related to reduced hearing.

The pair ICN18 (CC) and ICN19 (SMO) and the pair ICN16 (CC) and ICN12 (VIS) presented age-related correlation decrease during young adulthood and age-related anticorrelation increase in older age. Extrapolating the discussion in previous sections we could infer that the CC, SMO and VIS networks slowly switch from a “cooperative” to a “competitive” interaction, which may functionally result in difficulties to select and coordinate language, visual and sensorimotor tasks with increasing age.

Lastly, we observed age-related FNC correlation decrease between ICN17 (VIS) and ICN13 (CB), respectively located in the visual and cerebellar areas. Both areas share similar functions related to posture and balance. We could infer that the cerebellar and visual areas possibly lose the ability to ‘cooperate and compensate’ each other with age, resulting in lower postural stability.

**F. Supplementary ICN information: average TC spectra**

The average TC spectra (across all participants) of the 18 ICNs identified as belonging to six domains are presented in Fig. S10**.**


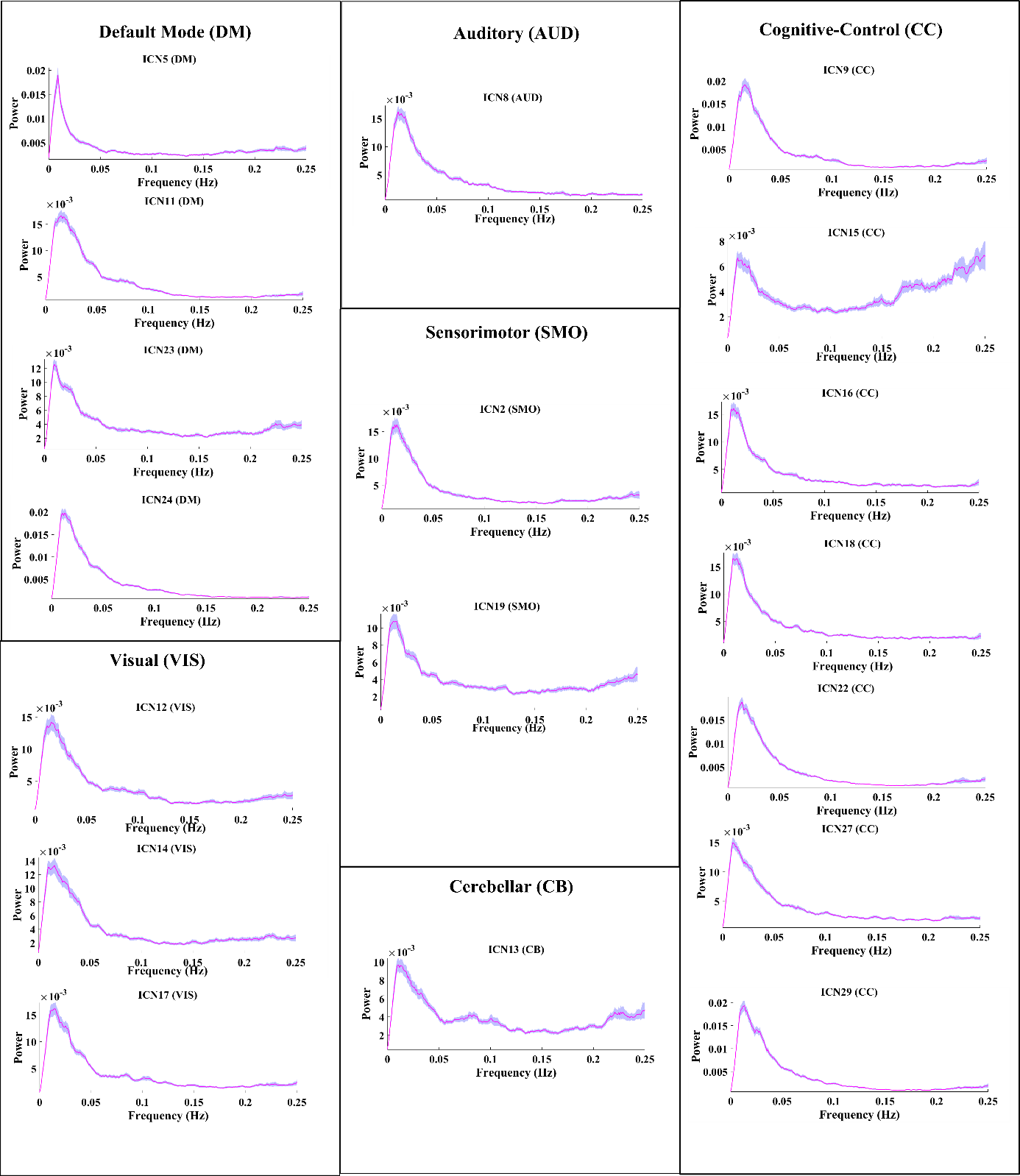


Figure S10 – TC spectra of the 18 intrinsic connectivity networks identified as belonging to functional domains.
